# Causes and consequences of variation in heterospecific pollen receipt in *Oenothera fruticosa*

**DOI:** 10.1002/ajb2.1720

**Published:** 2021-08-30

**Authors:** Gerard X. Smith, Mark T. Swartz, Rachel B. Spigler

**Affiliations:** ^1^ Department of Biology Temple University 1900 N. 12^th^ Street Philadelphia PA 19122 USA; ^2^ The Pennsylvania Department of Military and Veterans Affairs Fort Indiantown Gap National Guard Training Center Annville PA 17003 USA

**Keywords:** co‐flowering, floral neighborhood, flowering phenology, interspecific pollen transfer, intraspecific variation, Onagraceae, pollen load, pollination, pollinator effectiveness, seed set

## Abstract

**Premise:**

Heterospecific pollen transfer, the transfer of pollen between species, is common among co‐flowering plants, yet the amount of pollen received is extremely variable among species. Intraspecific variation in heterospecific pollen receipt can be even greater, but we lack an understanding of its causes and fitness consequences in wild populations.

**Methods:**

We examined potential drivers of variation in heterospecific pollen receipt in *Oenothera fruticosa*. We evaluated the relationship between heterospecific and conspecific pollen receipt and considered how visitation by different pollinator groups, local floral neighborhood composition, and flowering phenology affect the total amount and proportion of heterospecific pollen received. Finally, we tested whether variation in heterospecific pollen receipt translated into lower seed production.

**Results:**

Heterospecific pollen was ubiquitous on *O. fruticosa* stigmas, but the amount received was highly variable and unrelated to conspecific pollen receipt. Heterospecific pollen receipt depended on pollinator type, the proportion of nearby conspecific flowers, and flowering date. Significant interactions revealed that the effects of pollinator type and neighborhood were not independent, further contributing to variation in heterospecific pollen. Naturally occurring levels of heterospecific pollen were sufficient to negatively impact seed set, but large amounts of conspecific pollen counteracted this detrimental effect.

**Conclusions:**

Although selection could act on floral traits that attract quality pollinators and promote synchronous flowering in *O. fruticosa*, the risk of heterospecific pollen is equally dependent on local floral context. This work highlights how extrinsic and intrinsic factors contribute to intraspecific variation in heterospecific pollen receipt in wild plants, with significant fitness consequences.

Over 80% of angiosperms rely on animal pollinators for successful pollination (Ollerton et al., 2011). Yet because most plants share pollinators with several to a few dozen other co‐flowering species (Jordano, 1987; Waser et al., 1996), this success can be compromised by pollinator‐mediated competition among plant species (Brown et al., 2002; Lopezaraiza‐Mikel et al., 2007). Plants can compete directly for visitation by shared pollinators (Waser, 1978) or indirectly when shared pollinators transfer pollen between plant species, termed heterospecific pollen transfer (HPT; Morales and Traveset, 2008). Heterospecific pollen transfer is extremely common in co‐flowering communities, and its incidence and intensity vary markedly across species (McLernon et al., 1996; Fang and Huang, 2013; Arceo‐Gómez et al., 2016; Tur et al., 2016), with implications for the evolution of floral morphology, species divergence, and community structure (Moreira‐Hernández and Muchhala, 2019). Even as our understanding of the forces driving such interspecific variation grows (Ashman and Arceo‐Gómez, 2013; Arceo‐Gómez et al., 2016), investigations into the extent of variation in HPT within species and its underlying causes has scarcely begun (Moreira‐Hernández and Muchhala, 2019; Ashman et al., 2020). Indeed, few studies sample thoroughly enough to comment on within‐species variation in HPT (Arceo‐Gómez et al., 2018). Strikingly, one study indicates that intraspecific variation in stigmatic loads of heterospecific pollen (HP) can be greater than interspecific variation in HP loads (Arceo‐Gómez et al., 2016).

Most plant species are visited by a suite of generalist pollinator species (Waser et al., 1996). Indeed, studies examining the interactions within entire plant–pollinator communities have revealed broadly generalized interaction networks (Bascompte et al., 2003; Vázquez and Aizen, 2004). Visitation by the entire suite of pollinators need not be distributed evenly among individuals of a given plant species; instead, individuals—and even specific flowers on an individual—will be visited by some subset of pollinators (Bruckman and Campbell, 2014; Tur et al., 2014; Kuppler et al., 2016), contributing to intraspecific variation in pollination success. The effectiveness of any given pollinator depends in part on its morphology (e.g., body size), foraging behavior, and its visitation frequency (Sahli and Conner, 2007; King et al., 2013; Ballantyne et al., 2015; Koski et al., 2018; Page et al., 2019). Differences in these factors result in the common observation that floral visitors are not equally desirable as pollinators with respect to conspecific pollen (CP) deposition (King et al., 2013; Bruckman and Campbell, 2014; Koski et al., 2018). Pollinators are also known to vary in the quality of pollen they deliver (Herrera, 1987), but pollinator effectiveness with respect to HP deposition is less studied. Furthermore, fidelity to a given plant species within a foraging bout (floral constancy; Waser, 1986) is not necessarily widespread among pollinator taxa (Gross, 1992; Amaya‐Márquez, 2009). Consequently, pollinators should also vary in the amount of HP and/or the proportion of HP relative to CP they transfer. In fact, the relationship between HP and CP deposited within species may be driven in part by pollinator assemblage (Arceo‐Gómez et al., 2016).

The probability of HP receipt for any given plant might also be a problem of circumstance, specifically a plant's local neighborhood. Greater numbers of conspecific individuals within a local neighborhood are often associated with greater CP receipt (Ghazoul, 2005), at least up to a point (Benadi and Pauw, 2018), and can be more important than population‐level abundance measures in determining reproductive success (Roll et al., 1997; Spigler and Chang, 2008). However, the impacts of co‐flowering species in local floral neighborhoods on HP receipt are unclear (Morales and Traveset, 2008; Cariveau and Norton, 2009; Charlebois and Sargent, 2017; Ha and Ivey, 2017). For example, co‐flowering neighbors may facilitate pollinator visitation (Mitchell et al., 2009; Morales and Traveset, 2009; Tur et al., 2016), but negatively affect the purity of pollen loads transported (Brown et al., 2002; Bell et al., 2005), leading to a positive relationship between HP and CP receipt (Thomson et al., 2019). However, precisely because pollinators diverge in foraging patterns and those patterns are often governed by the abundance and distribution of flowering plants at small spatial scales (Feinsinger et al., 1991; Goulson, 2003; Ghazoul, 2005), the impact of the neighborhood on HP receipt likely varies with pollinator type.

Heterospecific pollen receipt might also change predictably across the flowering season or be stochastic. During the flowering period of a given species, large‐scale changes in the diversity, relative abundance, and composition of plant species in the flowering community typically occur (CaraDonna et al., 2017; Kantsa et al., 2018). Meanwhile, the flowering schedules of individual plants are not evenly distributed across the season, and early versus late flowering plants could have different pollination outcomes (Stone et al., 1998; Kitamoto et al., 2006; Chen et al., 2017). In addition, synchronous flowering of individuals within a population can be positively associated with successful pollination because it increases the absolute number of available mates and can boost signals to pollinators against the backdrop of the flowering community (Elzinga et al., 2007; Bartkowska and Johnston, 2014). If this results in pollinators temporarily specializing on a given species during its peak flower, individuals may not only receive more CP but less HP. On the other hand, if HP receipt is steady or stochastic as a consequence of the background “noise” of the community, then the proportion of HP may instead change across the season with variable CP deposition. Currently, we know little about intraseasonal variation in HP receipt and the relationship between HP receipt and flowering phenology.

Interest in HPT is driven in large part because it is anticipated to have major fitness consequences for plants, though current evidence is equivocal, revealing a mixture of negative and neutral fitness effects (Morales and Traveset, 2008; Ashman and Arceo‐Gómez, 2013; Moreira‐Hernández and Muchhala, 2019). Most of these studies represent experimental hand pollinations of flowers with mixes of CP and HP (Moreira‐Hernández and Muchhala, 2019) to test and isolate factors such as the arrival of HP relative to CP (Caruso and Alfaro, 2000; Bruckman and Campbell, 2016a), HP diversity (Ashman and Arceo‐Gómez, 2011), and HP donor identity (Arceo‐Gómez and Ashman, 2016; Arceo‐Gómez et al., 2019). Fewer have investigated the fitness consequences of naturally occurring levels of HP receipt in wild populations (Briggs et al., 2016; Suárez‐Mariño et al., 2019; Parra‐Tabla et al., 2020). The documentation of potential HP impacts on pollen tube development and extreme within‐species variability in HP receipt (Arceo‐Gómez et al., 2016, 2018; Briggs et al., 2016; Fang et al., 2019) suggests an opportunity for natural selection on traits that better allow plants to avoid HP receipt, but only if such variation is associated with fitness differences in natural populations.

In this study, we examined variation in HP receipt and its potential causes and consequences in the perennial *Oenothera fruticosa* L. (Onagraceae). Specifically, we asked the following sets of questions. (1) How much variation is there in HP receipt among *O. fruticosa* individuals, and what is the relationship between CP and HP receipt? (2) To what extent do pollinator identity, local floral neighborhood composition, and timing of flowering influence HP receipt? And, does the impact of local neighborhood depend on pollinator type? Finally, to address the significance of natural variation in HP receipt for plant fitness and its implications for microevolutionary dynamics, we asked (3) to what extent does this natural variation translate into variation in seed set?

## MATERIALS AND METHODS

### Study species and site

*Oenothera fruticosa* is an herbaceous, perennial native to eastern North America that occurs in open habitats such as fields and meadows. The species is hermaphroditic and blooms from June through July, producing showy yellow flowers that provide nectar as a floral reward. Individuals typically have 1 to 3 flowers open at a time; each flower contains an average of 150 ovules, opens in the morning, and lasts approximately 24 h. *Oenothera fruticosa* is self‐incompatible and thus requires outcross pollination from insects (Silander and Primack, 1978). Pollinated flowers develop into dehiscent capsules that mature around 3 weeks after pollination.

We conducted the study at Fort Indiantown Gap, an active National Guard training center, in Annville, Pennsylvania, United States. Approximately 88 ha of training areas and ranges on the base have been designated for researching the plant and animal species that thrive in periodically disturbed grassland habitats. In June 2019, we tagged 122 *O. fruticosa* plants distributed across a 12‐ha grassland that contains a diverse assemblage of >70 flowering plant species. Focal *O. fruticosa* plants were at least 2 m apart and tagged before flowering such that their blooming times were randomly distributed across the entire flowering season. From June to August, we randomly selected one flower per plant to follow from date of anthesis through to fruit set, quantifying pollinator visitation, pollen deposition, and seed production. We recorded diurnal insect visitation on each flower for approximately 1 h using high‐definition video cameras (Sony, San Diego, CA, USA) during pollinator activity (08:00–16:00 hours) on warm, sunny days, capturing a total of 130 h of film. We note that although many *Oenothera* species are visited by both diurnal and nocturnal floral visitors, the importance of these groups as pollinators varies across species (Rhodes et al., 2017; Antoń and Denisow, 2018; Krakos and Austin, 2021). Prior work has demonstrated that *O. fruticosa* is visited by diurnal pollinators including, butterflies, bees, and beetles, with no record of nocturnal visitors (Primack and Silander, 1975; Silander and Primack, 1978; Krakos and Austin, 2021). From the video footage, we identified pollinators and determined pollinator visitation rates of each, only considering insects that were observed contacting the reproductive structures of the flowers. Pollinators captured on film were almost exclusively bees (*Bombus, Ceratina, Halictus, Lasioglossum, Augochlorini*) and flies (*Milesia virginica, Toxomerus*); there were no visits from lepidopterans and only two visits from coleopterans, representing 0.7% of total visits. Therefore, we considered and quantified pollinator visitation rates of four functional groups: large bees, small bees, large flies, and small flies, consistent with pollinator groupings used in other studies of HP and CP deposition (Koski et al., 2015; Bruckman and Campbell, 2016b). Large and small bees were the most common (40.8% and 41.8%, respectively), followed by small flies (11.4%) and large flies (6.0%). Details about pollinator visitation rates, including mean visitation rates and proportion of flowers visited by each pollinator group can be found in Appendix S1.

### Composition of local floral neighborhoods

We quantified the composition of the local floral neighborhood within a 1‐m radius of each focal plant on the date visitation was filmed. This scale was chosen because it has been demonstrated to significantly influence pollinator visitation, conspecific pollen receipt, and seed production in a number of other grassland systems (e.g., Roll et al., 1997; Spigler and Chang, 2008; Cariveau and Norton, 2009). Within each neighborhood, we counted the number of open *O. fruticosa* flowers and open flowers (or floral units) of heterospecifics. We then calculated the proportion of conspecific flowers per neighborhood as the abundance of conspecific flowers divided by total flower number within the neighborhood. Floral units of heterospecifics were defined depending on the inflorescence structure of each species. For species with singular flowers, we were able to count individual flowers; for composite flowers or compact inflorescences with small flowers (e.g., asters), we considered each inflorescence as a floral unit. The most common co‐flowering species across the focal neighborhoods were *Securigera varia* (40% of heterospecific flowers), *Erigeron annuus* (22%), and *Leucanthemum vulgare* (19%); the remaining 20 species in bloom each represented 5% or less, though they still occurred in up to 37% of neighborhoods (Appendix S2).

### Pollen deposition

We collected stigmas of all filmed flowers the day after filming, when flowers were wilted, using clean forceps and stored them in 70% ethanol until processing. Because *O. fruticosa* has relatively large stigmas, we used an acetolysis processing step (Kearns and Inouye, 1993) to remove stigmatic tissue and retain only pollen grains. We mounted post‐acetolysis pollen samples in ethanol and examined under a compound light microscope (Nikon, Tokyo, Japan) to count conspecific and heterospecific grains. Pollen grains of *O. fruticosa* have a unique triangular shape and are especially large (>100 µm long), making them easily distinguishable from pollen grains of all other species in the community. We considered that pollen grains could be lost during wash steps of the acetolysis procedure. We captured the wash and quantified pollen lost in these steps for a subset of samples. The amount of pollen grains lost was minimal (mean pollen lost <1% per sample), such that we effectively have complete counts per stigma.

### Fruit collection and seed set

Finally, we collected mature fruits approximately 3 weeks after flowering and stored them in coin envelopes until processing. In total, we were able to collect 93 fruits from the 122 flowers that were filmed. Some of the fruits went missing in the field or experienced herbivory and were not included in the analyses. Each fruit was carefully examined with a dissecting microscope to count fully developed seeds and unfertilized ovules. We calculated seed set as the number of developed seeds divided by total ovule number (the sum of developed seeds and unfertilized ovules).

### Statistical analyses

We evaluated the relationship between HP and CP deposition. We considered both linear and nonlinear relationships given these patterns may reflect different ecological mechanisms (Arceo‐Gómez et al., 2016). We evaluated separate general linear models including a linear‐response model (nontransformed data), log‐linear model (HP was log‐transformed), and log‐log model (HP and CP were log‐transformed), the latter two of which would indicate a nonlinear relationship between HP and CP. Due to a single zero‐value for HP grains, we added one to HP grain number before log‐transformation. We note that we also considered generalized linear models: a Poisson error distribution, which proved to fit poorly based on residuals and the dispersion parameter (results not shown), and a negative binomial, which proved better but with qualitatively similar results to the general linear models (results not shown).

We evaluated the contributions of different pollinator groups, local floral neighborhood composition, and flowering phenology to both the number and proportion of HP grains received using a linear model and a generalized linear model, respectively. In the former, we used the square root of HP grain number per stigma as the response variable to conform to model assumptions. In the latter, we modeled the proportion of HP received with a binomial error distribution and logit link function weighted by the total number of grains per stigma. Six of the flowers filmed were excluded from these analyses because of partially missing data (*N* = 116). In both models, we included visitation by each of the four main pollinator groups (large bees, small bees, large flies, and small flies) as independent binary predictor variables because of extreme overdispersion in the distribution of pollinator visitation rates. We also included as predictor variables: proportion of neighborhood conspecific flowers and date (Julian day) of flowering. Both predictors were *z*‐transformed to allow for comparisons between their dissimilar scales. Finally, to test whether the impact of pollinators is context‐dependent, we included interactions between each visitor group variable and proportion of conspecific flowers. However, to maximize power of our analyses to detect main effects, interactions were retained only when significant.

To test whether increasing HP receipt translates to lower reproductive output of *O. fruticosa*, we used a generalized linear model to model seed set following a binomial error distribution, weighted by total ovule number. This model included fixed effects for CP grain number, HP grain number, and their interaction. Julian day was included as a covariate to account for differences in seed set across the season. Each predictor was *z*‐transformed to allow for comparisons between their dissimilar scales. All analyses, unless otherwise noted, were conducted using R 4.0.2 (R Core Team, 2020).

## RESULTS

### Conspecific and heterospecific pollen receipt

**Figure 1:**
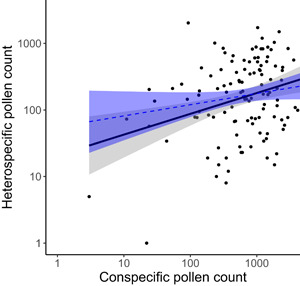
Linear regression of the relationship between heterospecific pollen and conspecific pollen deposited on *Oenothera fruticosa* stigmas. Both heterospecific pollen counts and conspecific pollen counts were log‐transformed for analysis and are plotted here on a log scale. Shading indicates 95% confidence intervals for regression line. Black solid line and gray shading are for the entire data set (*N* = 122); blue dashed line and blue shading are for the data set with two overly influential points removed (*N* = 120)

Deposition of HP on *O. fruticosa* flowers at our site was ubiquitous: all but one of the 122 flowers we examined received HP. On average, *O. fruticosa* flowers received 319.9 (± 362.8 SD; range 0–2030) HP grains compared to an average of 998.7 (±938.9 SD; range 2–5016) CP grains. Both were highly variable; when scaled by the mean, HP receipt was more so (CV = 113) than CP receipt (CV = 94). Mean proportion HP per stigma was 0.30 (±0.25 SD) and highly variable as well (range 0–0.98). We did not detect a linear relationship between HP and CP grain number (i.e., untransformed data; *F* = 0.89, df = 1, *P* = 0.35) nor a significant log‐linear relationship (*F* = 3.01, df = 1, *P* = 0.09). The log‐log relationship between HP and CP grain number was significant (*F* = 8.10, df = 1, *P* = 0.005), but this relationship was weak (*R* ^2^ = 0.06; Figure 1) and hinged on two data points (removed: *F* = 1.34, df = 1, *P* = 0.25).

**Figure 2:**
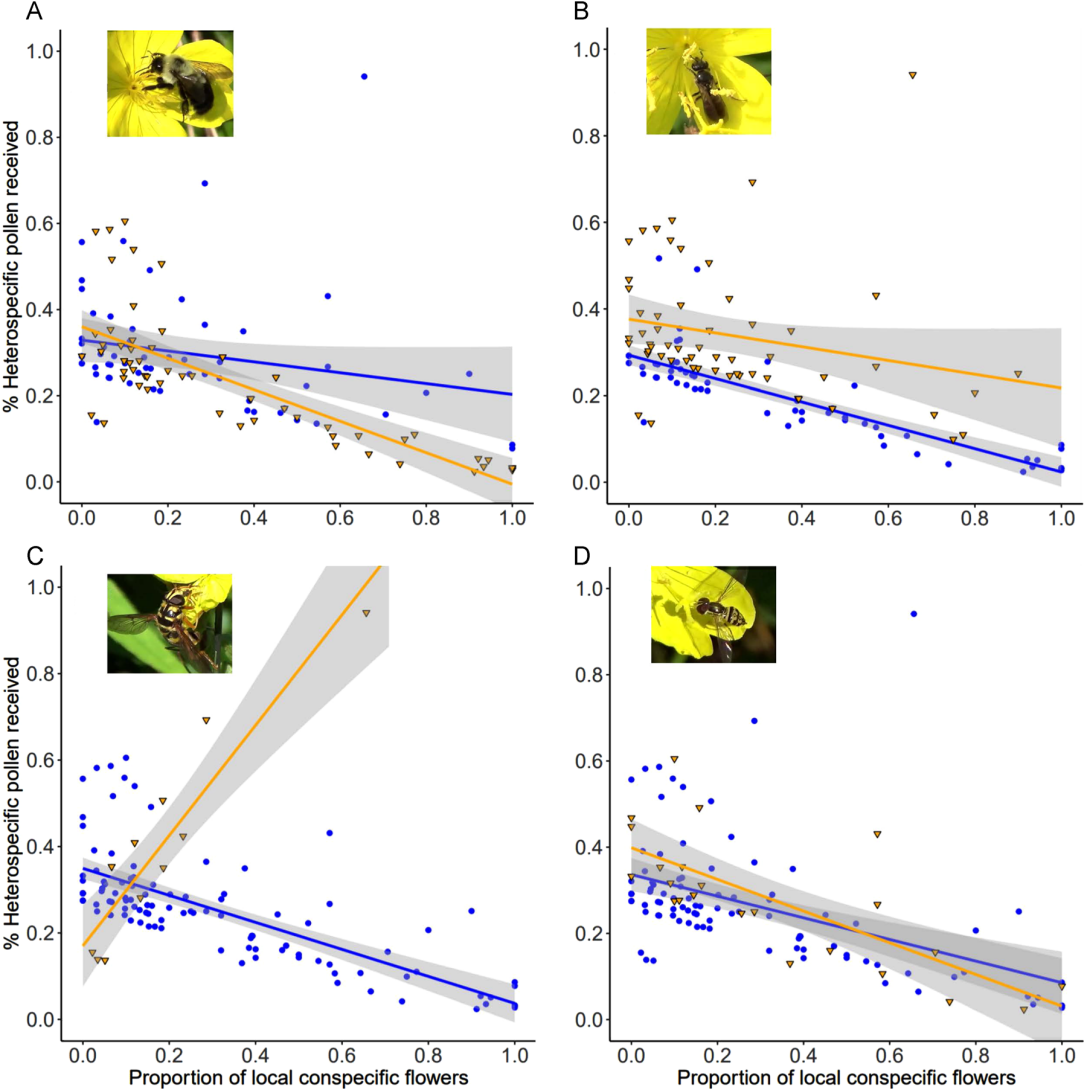
Scatterplots of the proportion heterospecific pollen received by the proportion of conspecific flowers in local floral neighborhoods. *N* = 116. This relationship is mediated by the visitation of various pollinator groups: (A) large bees, (B) small bees, (C) large flies, and (D) small flies. Yellow triangles and lines represent flowers that were visited by each group; blue circles and lines represent flowers not visited by each group. Shading indicates 95% confidence intervals of each line

Both the number of HP grains and proportion HP grains decreased significantly as the proportion of conspecific flowers increased in the neighborhood (Table 1). However, for proportion HP, significant interactions between proportion conspecific flowers and each pollinator group revealed that the effects of neighborhood composition and pollinator type depended on each other (Table 1, Figure 2). The negative relationship between proportion HP received and proportion local conspecific abundance was significantly steeper when plants were visited by large bees and small flies (Figure 2A, D). For flowers visited by large bees, this amounted to similar proportions of HP deposited in neighborhoods where conspecific flowers ranged from low density to absent, regardless of whether large bees visited (Figure 2A). Yet as conspecific density increased, visitation by large bees becomes increasingly important, such that the proportion of HP received was significantly lower for plants visited by large bees. Plants visited by small bees consistently received greater proportions of HP regardless of conspecific floral density. In this case, the interaction arises because the benefit of conspecific neighbors was substantially weakened for plants visited by small bees, i.e., proportion HP deposited was relatively constant under small bee visitation (Figure 2B). Surprisingly, visitation by large flies reversed the relationship between proportion HP receipt and percentage conspecific flowers (Figure 2C). This pattern remains significant even when we removed the data point representing the highest proportion HP received under large fly visitation (results not shown). In contrast to the results for proportion HP deposition, only small bee visitation significantly influenced the number of HP grains per stigma, increasing HP deposition independently of neighborhood composition (Table 1).

**Table 1:** Results from linear and generalized linear models on the effects of insect visitation, local neighborhood composition, and Julian day on heterospecific pollen receipt in *Oenothera fruticosa*

	Response variables						
	Count of HP received				Proportion of HP received		
Fixed effects	df	Estimate	*F*	*P*	Estimate	χ^2^	*P*
Large bee visitation	1	0.628	0.151	0.698	**–0.167**	**155.80**	**<0.001**
Small bee visitation	1	**4.149**	**5.911**	**0.017**	**0.341**	**623.14**	**<0.001**
Large fly visitation	1	–3.502	1.342	0.249	**0.642**	**104.30**	**<0.001**
Small fly visitation	1	0.933	0.213	0.645	0.033	3.46	0.063
%Con	1	**–2.696**	**12.11**	**0.001**	**–0.401**	**758.69**	**<0.001**
Julian day	1	–1.710	2.607	0.109	**0.442**	**1788.88**	**<0.001**
Large bee × %Con	1	—	—	—	**–0.327**	**464.34**	**<0.001**
Small bee × %Con	1	—	—	—	**0.198**	**177.75**	**<0.001**
Large fly × %Con	1	—	—	—	**2.179**	**484.12**	**<0.001**
Small fly × %Con	1	—	—	—	**–0.178**	**102.78**	**<0.001**

*Notes:* %Con is the proportion of conspecific flowers in local floral neighborhoods. The heterospecific pollen count model uses square‐root‐transformed counts of heterospecific pollen as a response with a Gaussian distribution. The proportion heterospecific pollen model uses a binomial distribution. Visitation by each pollinator group is coded as a binary predictor variable. %Con and Julian day were *z*‐transformed. Significant model parameters are shown in bold. *N* = 116. Only significant interactions were retained for final models.

**Figure 3:**
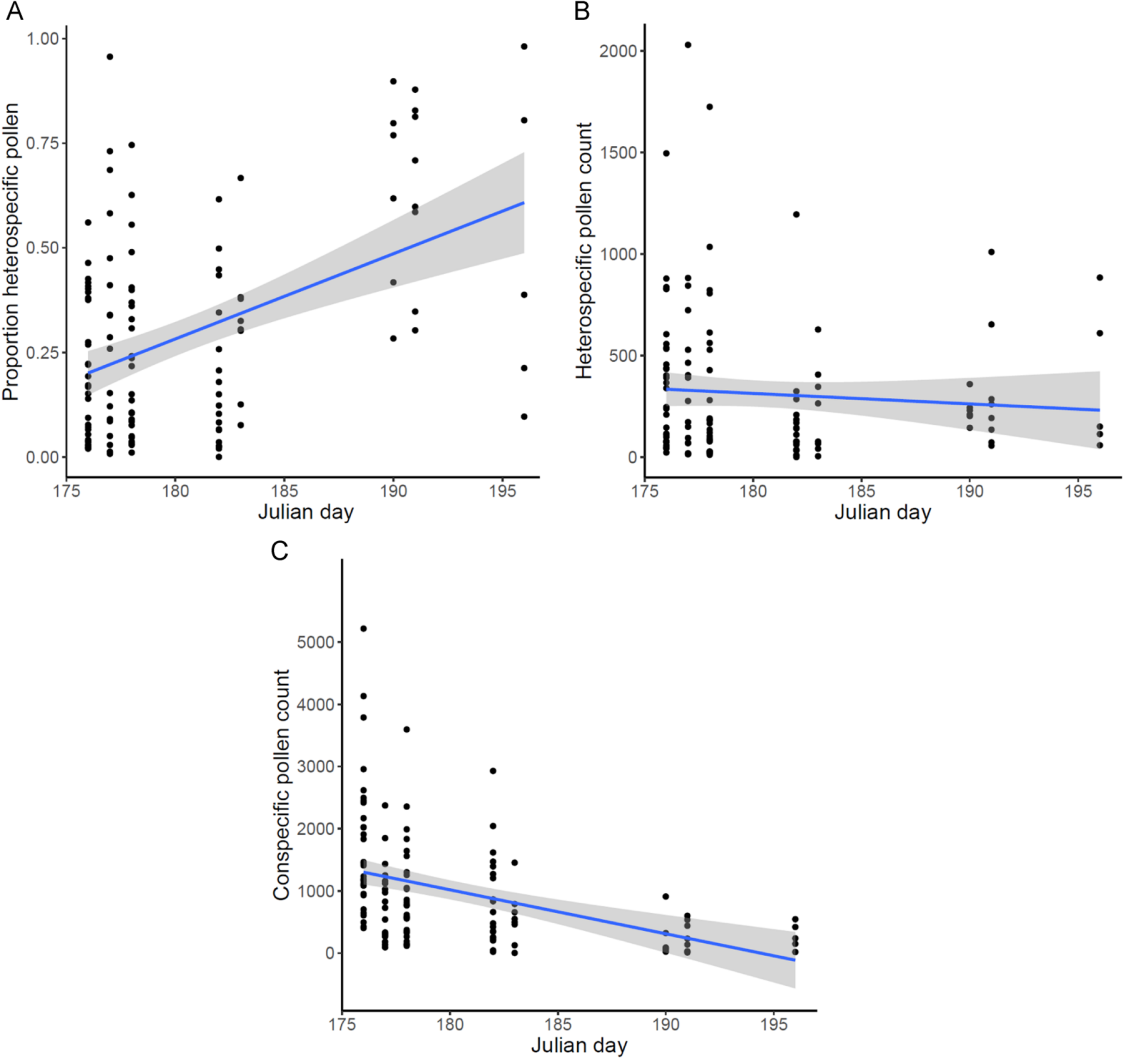
Scatterplots of the proportion of heterospecific pollen (A), count of heterospecific pollen (B), and count of conspecific pollen (C) for individual *Oenothera fruticosa* flowers through the season. *N* = 122. Blue lines depict linear regressions with 95% confidence intervals

Additionally, we found that flowers opening later in the season received significantly greater proportions of HP (Table 1, Figure 3A). The number of HP grains, however, was not significantly affected by phenology (Table 1, Figure 3B). Thus, despite constant HP receipt across the season, the proportion HP increased because CP deposition declined later in the season (*F* = 25.80, df = 1, *P* < 0.001; Figure 3C).

### The effect of pollen receipt on seed production

**Figure 4:**
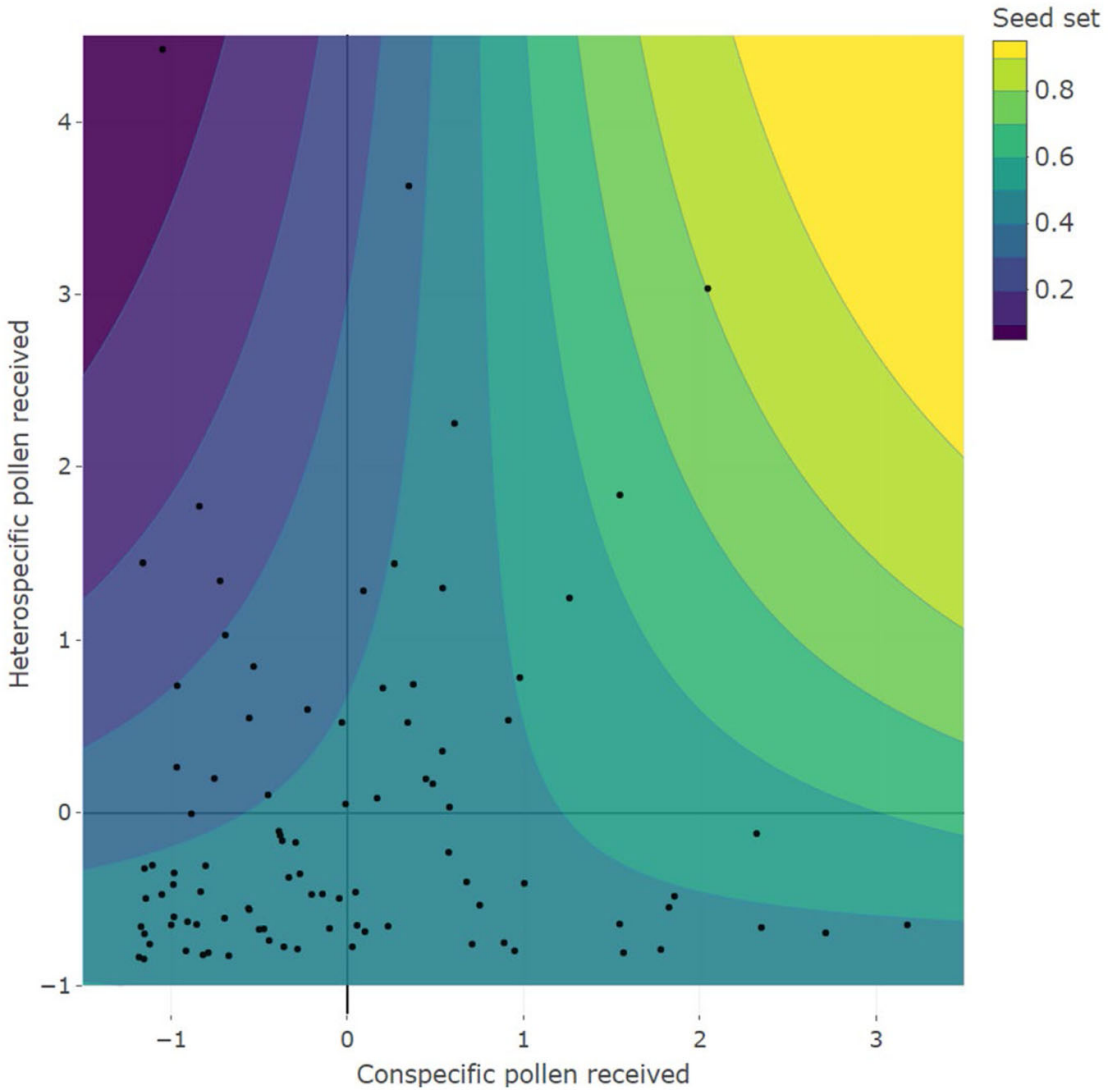
Contour plot representing predicted seed set values for given amounts of heterospecific and conspecific pollen deposited. *N* = 93. Pollen counts are *z*‐transformed; 0 represents the mean observed value on each axis. Black dots represent data points used in the model. The detrimental fitness impact of receiving heterospecific pollen occurs in stigmas that received fewer than the mean value of conspecific pollen grains, but disappears for moderate/high CP values

Seed set was highly variable (39.3% ± 27.5%, mean ± SD) and significantly influenced by the composition of pollen deposited and phenology (Table 2). We found that seed set was negatively related to the number of HP grains per stigma, positively related to CP grain number, and declined across the season (Table 2). However, there was also a significant interaction between HP and CP deposition (Figure 4). In particular, the negative impact of HP deposition was strongest at low levels of CP, below the mean (*z*‐scaled mean = 0 in Figure 4) and then began to dissipate as the number of CP grains on the stigma increased. At the highest levels of CP receipt, there even appeared to be a positive impact of HP, though we caution that this area of the contour map was not well supported, based on a few data points of excessively high CP and HP. With respect to HP loads, we saw that greater CP receipt can rescue seed set of plants with heavy HP. Ultimately, our data support that the proportion of heterospecific and the total number of CP and HP grains received influences seed set.

**Table 2:** Results from generalized linear model on the effects of pollen receipt and Julian day on seed set in *Oenothera fruticosa*

	Response variable			
	Seed set			
Fixed effects	df	Estimate	χ^2^	*P*
CP count	1	**0.226**	**57.62**	**<0.001**
HP count	1	**−0.194**	**38.42**	**<0.001**
Julian day	1	**−0.270**	**111.84**	**<0.001**
CP count × HP count	1	**0.289**	**175.77**	**<0.001**

*Notes:* CP count and HP count are counts of conspecific and heterospecific pollen deposited on stigmas, respectively. This model uses seed set as a response with a binomial distribution. All predictor variables were *z*‐transformed. Significant model parameters are shown in bold. *N* = 93.

## DISCUSSION

Our study identified extrinsic and intrinsic factors shaping intraspecific variation in HP receipt. We revealed how the interaction between pollinator identity and local floral neighborhood, together with a plant's flowering phenology, affect HP deposition on *O. fruticosa* flowers. HP receipt was largely independent of CP receipt and sufficient to negatively impact seed set. Our study joins only one other in demonstrating the consequences of HP loads for seed production in wild populations (Briggs et al., 2016). We discuss our results and their implications for natural selection to avoid HP receipt and its constraints.

### Intraspecific variation in heterospecific pollen receipt

Pollinators notoriously vary in their effectiveness at depositing CP (Herrera, 1989; King et al., 2013; Ballantyne et al., 2015), but their effectiveness with respect to CP need not correlate with their probability of depositing HP (Mitchell et al., 2009; Arceo‐Gómez et al., 2016; Ashman et al., 2020). In general, we found that HP and CP deposition stigmas were not related—or at best only weakly so—in *O. fruticosa*. Arceo‐Gómez et al. (2016) suggested that such independence could arise if one or few high‐quality pollinators deliver nearly pure CP loads, while HP is brought stochastically by ineffective pollinators. The number of HP grains received by *O. fruticosa* varied only with visitation by small bees, but visitation by all pollinator groups influenced the *proportion* of HP received. Thus, all these pollinator groups likely bring varying amounts of CP with relatively consistent HP loads, resulting in a gradient of pollinator effectiveness. Likewise, Fang et al. (2019) suggest that species receiving high average HP loads are likely to be visited by pollinators that carry relatively high, consistent amounts of HP.

Nearly half of the visits to *O. fruticosa* flowers in the population under study were by small bees, which are inefficient pollinators in some systems (Gorenflo et al., 2017; Koski et al., 2018; Konzmann et al., 2019). Indeed, two previous studies of pollinator importance in other *Oenothera* species found that small bees functioned as poor pollinators or pollen thieves (Artz et al., 2010; Rhodes et al., 2017). We showed that both the number and proportion of HP were greater for *O. fruticosa* flowers visited by small bees. In contrast, visitation by large bees, which comprised a similar proportion of visits as small bees, had no influence on the number of HP grains but was associated with a lower proportion of HP grains. Thus, the large bees deposited higher amounts or proportions of CP, relative to the less effective pollinators in our system. The comparison of HP loads from small and large bees was consistent with this interpretation; flowers that were visited only by small bees during our observation periods received slightly more HP and slightly less CP on average than those visited solely by large bees, and the proportion HP received was significantly lower for flowers visited by only large bees (χ^2^ = 1419.1, df = 1, *P* < 0.001). The well‐documented floral constancy of *Bombus* pollinators during foraging bouts may be responsible for this effect (Goulson, 2003). Indeed, multiple studies have reported that large amounts of CP are lost when pollinators switch between species during a foraging bout (Flanagan et al., 2009; Muchhala and Thomson, 2012). For constant pollinators, the transfer of CP grains may be more strongly influenced by pollinator behavior than the transfer of HP grains, which appears to be a more consistent by‐product of pollinator sharing. Nocturnal visitors have not been documented previously for *O. fruticosa*, but if present in this population, could also have contributed to HPT. Nevertheless, though we cannot empirically exclude their importance, we did see strong patterns of HPT related to diurnal visitors alone.

We further showed that HP deposition in *O. fruticosa* was context dependent, influenced by a plant's local floral neighborhood and the way pollinators interact with this neighborhood. Individuals in local neighborhoods with high proportions of conspecific flowers received fewer HP grains and a lower proportion of HP relative to CP. The strength and even the direction of impact of floral neighborhood, however, was mediated by pollinator group. Theoretical models predict changes in pollinator foraging behavior with changes in the relative densities of species in mixed plant assemblages (Goulson, 1994; Kunin and Iwasa, 1996). We found a steeper decrease in the proportion of HP received per increase in proportion of local conspecific flowers for flowers visited by large bees and small flies compared to flowers they did not visit. The difference in slope was strongest for large bees, which might be expected for constant foragers, either because the absolute increase in local CP availability in neighborhoods with greater *O. fruticosa* relative abundance enables constant pollinators to bring in greater amounts of CP relative to HP or because their constancy (Waser, 1986) or preference (Smithson, 2001) increases with *O. fruticosa* density. The latter cases would lead to these pollinators visiting *O. fruticosa* disproportionately as its floral density increases. Flowers visited by small bees exhibited a weaker negative relationship between proportion of HP received and proportion of conspecifics in their floral neighborhoods. In neighborhoods with higher *O. fruticosa* abundance, this relationship translates to a greater proportion of HP on flowers visited by small bees compared to those that were not. This pattern is again consistent with small bees depositing pollen loads generally of poorer purity (more HP) and suggests that small bees foraging behavior is influenced less by the composition of local floral neighborhoods than that of other visitors.

Surprisingly, when large flies visited, the proportion of HP increased as local conspecific relative abundance increased. We suggest caution in interpreting this curious finding, given visits by *M. virginica* were relatively rare during our study. Still, we speculate that such a pattern could arise if large flies exhibit negative‐frequency‐dependent foraging (Smithson, 2001) on *O. fruticosa*, i.e., visitation to *O. fruticosa* is inversely related to *O. fruticosa* abundance. Negative‐frequency‐dependent foraging can occur when rarer pollinators must compete with more common pollinators for access to the more common resource, assuming that other plant species are equally rewarding (Eckhart et al., 2006). Alternatively, we observed the large fly, *M. virginica*, consuming pollen from *O. fruticosa* anthers and even the stigma. If instead these pollinators increase visitation to *O. fruticosa* when *O. fruticosa* is abundant, they might remove more CP from stigmas while still depositing HP. Consideration of neighbor identity might help unpack the interactions causing patterns involving large flies as well as those of the other pollinator groups. For example, co‐flowering species with similar floral morphologies and colors (Stout et al., 1998; Bell et al., 2005; Liao et al., 2011) or substantially more attractive and rewarding flowers (Randle et al., 2018; Thomson et al., 2019) may encourage pollinators to switch between species more frequently during a given foraging bout and transfer more pollen between species.

Timing of flowering is well known to influence HPT dynamics and competition for pollinators more generally among species. In fact, these dynamics can lead to character divergence and shape community structure (Mosquin, 1971; Pleasants, 1980; Rathcke, 1983). Our results illustrate the significant role of flowering time in determining intraspecific variation in HP receipt. Specifically, the proportion of HP found on *O. fruticosa* flowers increased predictably with flowering date across the season, and the strength of this effect was as great as the effect of local neighborhood. This increase in the proportion of HP occurred even though the number of HP grains received did not change with flowering time or at best declined weakly, because CP receipt declined significantly across the same period. Thus, as the flower season progresses, fewer individuals remained in bloom, reducing the amount of CP available for transport in the population. This shortage of CP and the consistent receipt of HP lead to increased proportions of HP for late‐flowering individuals. Additionally, lower CP deposition rates at the end of the season were not due to decreased pollinator visitation (*F* = 14.88, df = 1, *P* < 0.001; *R* ^2 ^= 0.11). Rather, the seasonal change in proportion HP deposition highlights the importance of flowering synchronously against a backdrop of steady HP arrival. Synchronous flowering increases the amount of CP available for transport in the population (Méndez and Díaz, 2001; Elzinga et al., 2007). Since *O. fruticosa* population‐level flower abundance was positively skewed and leptokurtic in our study population (Smith et al., 2021), early flowering *O. fruticosa* plants were likely more synchronous with the population. Thus, synchronous flowering can not only benefit plants by increasing CP receipt, but also by reducing the proportion of HP received.

### Fitness consequences of heterospecific pollen receipt

Both the number and proportion of HP on stigmas influenced seed production, demonstrating a clear detrimental fitness effect of HP receipt in *O. fruticosa*. Although the fitness impacts of receiving HP have been shown in many hand‐pollination experiments, recent work has shown similar negative impacts on CP tube growth (Suárez‐Mariño et al., 2019; Parra‐Tabla et al., 2020) and seed set (Briggs et al., 2016) in wild plant populations. There are many mechanisms by which HP can cause reproductive interference including stigma clogging (Galen and Gregory, 1989), pollen allelopathy (Murphy and Aarssen, 1995), style clogging (Randall and Hilu, 1990), and ovule usurping (Burgess et al., 2008). We suspect that interference by HP in our system likely took place on the stigmatic surface, given that HP tube growth in the style and via ovule usurpation is unlikely between distantly related species (Moreira‐Hernández and Muchhala, 2019) and that no confamilial species bloomed during our study. Perhaps key is that we found that the interaction between the amount of CP and HP influences seed set; seed set was the lowest for flowers that received low amounts of CP and high amounts of HP. Low CP deposition alone, however, is unlikely the cause because seed set is relatively constant across the range of CP amounts seen in our study, so long as HP is also low (Figure 4). The proportion of HP on stigmas has been found to influence seed set in other systems as well (Thomson et al., 1981; Briggs et al., 2016) and is more suggestive of neutral dilution or allelopathic effects of HP that reduce pollen germination rates and pollen tube growth of CP (Suárez‐Mariño et al., 2019; Parra‐Tabla et al., 2020). High levels of CP may be able to effectively neutralize the negative impacts of HP. Although detailed experiments are needed to identify the mechanism, our results suggest that there may be a threshold level of CP receipt *O. fruticosa* flowers can reach to avoid the detrimental impacts of HP receipt.

Given the substantial fitness impacts of HP, the question is whether and how selection could act for plants to avoid the detrimental impacts of HP receipt. It has been hypothesized that species may adopt alternative strategies of HP avoidance or tolerance to minimize the negative effects of HP receipt (Ashman and Arceo‐Gómez, 2013; Fang et al., 2019). Extrinsic factors such as a plant's local floral neighborhood cannot be controlled, which combined with a constant background threat of HP deposition seen in our study, limits the ability of *O. fruticosa* to avoid HP. Instead, our findings suggest that the best defense against HP in *O. fruticosa* is to increase CP deposition. We expect to see selection for earlier flowering synchrony, which was associated with receiving lower proportions of HP relative to CP. In addition, although we did not measure stigma sizes in this study, if the large stigmas of *O. fruticosa* allow it to tolerate HP, as suggested for other species (Montgomery and Rathcke, 2012; Arceo‐Gómez and Ashman, 2014), then selection could also favor plants with larger stigmas in this population. Finally, there is the opportunity for selection on floral traits in *O. fruticosa* that influence attraction of pollinators bringing low proportions of CP. For instance, Kuppler et al. (2016) found the composition of pollinators visiting individual *Sinapis arvensis* plants varied with floral phenotypes and influenced reproductive output. Other studies also found that pollinator groups discriminate between individuals based on floral traits like flower color (Briggs et al., 2018), floral scents (Parachnowitsch et al., 2012), and flower size (Conner and Rush, 1996; Mothershead and Marquis, 2000). Individual *O. fruticosa w*ould benefit from attracting fewer small bees because they bring more HP grains and higher proportions of HP.

## CONCLUSIONS

Our study highlights how pollinators interact with the composition of floral neighborhood at local spatial scales to influence intraspecific variation in heterospecific pollen receipt. By flowering earlier, and perhaps more synchronously, *O. fruticosa* can increase CP receipt and so offset the cost of constant HP receipt across the flowering season. The combination of intrinsic and extrinsic forces influencing HP receipt can help explain why heterospecific pollen is so ubiquitous among flowering plants (Fang and Huang, 2013; Arceo‐Gómez et al., 2016; Tur et al., 2016), despite its detrimental impacts.

## AUTHOR CONTRIBUTIONS

G.X.S. and R.B.S. conceived of and designed the study. G.X.S. conducted the study with help from M.T.S. G.X.S. analyzed the data, and G.X.S. and R.B.S. wrote the paper with contributions from M.T.S.

## Supporting information


**Appendix S1.** Table containing details about pollinator visitation rates, including mean visitation rates and proportion of flowers visited by each group.

**Appendix S2.** Table containing the counts and presence of co‐flowering heterospecific plant species across all floral neighborhoods.

## Data Availability

Data used for this study available from the Dryad Digital Repository: https://doi.org/10.5061/dryad.hqbzkh1g9 (Smith Jr. et al., 2021).

## References

[ajb21720-bib-0001] Amaya‐Márquez, M. 2009. Floral constancy in bees: a revision of theories and a comparison with other pollinators. Revista Colombiana de entomología 35: 206–216.

[ajb21720-bib-0002] Antoń, S. , and B. Denisow . 2018. Pollination biology and breeding system in five nocturnal species of Oenothera (Onagraceae): reproductive assurance and opportunities for outcrossing. Plant Systematics and Evolution 304: 1231–1243.

[ajb21720-bib-0003] Arceo‐Gómez, G. , L. Abdala‐Roberts , A. Jankowiak , C. Kohler , G. A. Meindl , C. M. Navarro‐Fernández , V. Parra‐Tabla , et al. 2016. Patterns of among‐ and within‐species variation in heterospecific pollen receipt: the importance of ecological generalization. American Journal of Botany 103: 396–407. 26507115 10.3732/ajb.1500155

[ajb21720-bib-0004] Arceo‐Gómez, G. , C. Alonso , T.‐L. Ashman , and V. Parra‐Tabla . 2018. Variation in sampling effort affects the observed richness of plant–plant interactions via heterospecific pollen transfer: implications for interpretation of pollen transfer networks. American Journal of Botany 105: 1601–1608. 30168577 10.1002/ajb2.1144

[ajb21720-bib-0005] Arceo‐Gómez, G. , and T.‐L. Ashman . 2014. Coflowering community context influences female fitness and alters the adaptive value of flower longevity in Mimulus guttatus. American Naturalist 183: E50–E63. 10.1086/674358 24464206

[ajb21720-bib-0006] Arceo‐Gómez, G. , and T.‐L. Ashman . 2016. Invasion status and phylogenetic relatedness predict cost of heterospecific pollen receipt: implications for native biodiversity decline. Journal of Ecology 104: 1003–1008.

[ajb21720-bib-0007] Arceo‐Gómez, G. , R. L. Kaczorowski , C. Patel , and T.‐L. Ashman . 2019. Interactive effects between donor and recipient species mediate fitness costs of heterospecific pollen receipt in a co‐flowering community. Oecologia 189: 1041–1047. 30877578 10.1007/s00442-019-04379-z

[ajb21720-bib-0008] Artz, D. R. , C. A. Villagra , and R. A. Raguso . 2010. Spatiotemporal variation in the reproductive ecology of two parapatric subspecies of Oenothera cespitosa (Onagraceae). American Journal of Botany 97: 1498–1510. 21616904 10.3732/ajb.1000086

[ajb21720-bib-0009] Ashman, T.‐L. , C. Alonso , V. Parra‐Tabla , and G. Arceo‐Gómez . 2020. Pollen on stigmas as proxies of pollinator competition and facilitation: complexities, caveats and future directions. Annals of Botany 125: 1003–1012. 31985008 10.1093/aob/mcaa012 PMC7262468

[ajb21720-bib-0010] Ashman, T.‐L. , and G. Arceo‐Gómez . 2011. Heterospecific pollen deposition: does diversity alter the consequences? New Phytologist 192: 738–746. 21777248 10.1111/j.1469-8137.2011.03831.x

[ajb21720-bib-0011] Ashman, T.‐L. , and G. Arceo‐Gómez . 2013. Toward a predictive understanding of the fitness costs of heterospecific pollen receipt and its importance in co‐flowering communities. American Journal of Botany 100: 1061–1070. 23624924 10.3732/ajb.1200496

[ajb21720-bib-0012] Ballantyne, G. , K. C. R. Baldock , and P. G. Willmer . 2015. Constructing more informative plant–pollinator networks: visitation and pollen deposition networks in a heathland plant community. Proceedings of the Royal Society, B, Biological Sciences 282: 20151130. 10.1098/rspb.2015.1130 PMC4571695 26336181

[ajb21720-bib-0013] Bartkowska, M. P. , and M. O. Johnston . 2014. The sexual neighborhood through time: competition and facilitation for pollination in Lobelia cardinalis. Ecology 95: 910–919. 24933810 10.1890/13-0447.1

[ajb21720-bib-0014] Bascompte, J. , P. Jordano , C. J. Melian , and J. M. Olesen . 2003. The nested assembly of plant–animal mutualistic networks. Proceedings of the National Academy of Sciences 100: 9383–9387. 10.1073/pnas.1633576100 PMC170927 12881488

[ajb21720-bib-0015] Bell, J. M. , J. D. Karron , and R. J. Mitchell . 2005. Interspecific competition for pollination lowers seed production and outcrossing in Mimulus ringens. Ecology 86: 762–771.

[ajb21720-bib-0016] Benadi, G. , and A. Pauw . 2018. Frequency dependence of pollinator visitation rates suggests that pollination niches can allow plant species coexistence I. Journal of Ecology 106: 1892–1901.

[ajb21720-bib-0017] Briggs, H. M. , L. M. Anderson , L. M. Atalla , A. M. Delva , E. K. Dobbs , and B. J. Brosi . 2016. Heterospecific pollen deposition in Delphinium barbeyi: linking stigmatic pollen loads to reproductive output in the field. Annals of Botany 117: 341–347. 26658101 10.1093/aob/mcv175 PMC4724048

[ajb21720-bib-0018] Briggs, H. M. , S. Graham , C. M. Switzer , and R. Hopkins . 2018. Variation in context‐dependent foraging behavior across pollinators. Ecology and Evolution 8: 7964–7973. 30250676 10.1002/ece3.4303 PMC6144987

[ajb21720-bib-0019] Brown, B. J. , R. J. Mitchell , and S. A. Graham . 2002. Competition for pollination between an invasive species (purple loosestrife) and a native congener. Ecology 83: 2328–2336.

[ajb21720-bib-0020] Bruckman, D. , and D. R. Campbell . 2014. Floral neighborhood influences pollinator assemblages and effective pollination in a native plant. Oecologia 176: 465–476. 25047026 10.1007/s00442-014-3023-6

[ajb21720-bib-0021] Bruckman, D. , and D. R. Campbell . 2016a. Pollination of a native plant changes with distance and density of invasive plants in a simulated biological invasion. American Journal of Botany 103: 1458–1465. 27539258 10.3732/ajb.1600153

[ajb21720-bib-0022] Bruckman, D. , and D. R. Campbell . 2016b. Timing of invasive pollen deposition influences pollen tube growth and seed set in a native plant. Biological Invasions 18: 1701–1711.

[ajb21720-bib-0023] Burgess, K. S. , M. Morgan , and B. C. Husband . 2008. Interspecific seed discounting and the fertility cost of hybridization in an endangered species. New Phytologist 177: 276–284. 17944826 10.1111/j.1469-8137.2007.02244.x

[ajb21720-bib-0024] CaraDonna, P. J. , W. K. Petry , R. M. Brennan , J. L. Cunningham , J. L. Bronstein , N. M. Waser , and N. J. Sanders . 2017. Interaction rewiring and the rapid turnover of plant–pollinator networks. Ecology Letters 20: 385–394. 28156041 10.1111/ele.12740

[ajb21720-bib-0025] Cariveau, D. P. , and A. P. Norton . 2009. Spatially contingent interactions between an exotic and native plant mediated through flower visitors. Oikos 118: 107–114.

[ajb21720-bib-0026] Caruso, C. M. , and M. Alfaro . 2000. Interspecific pollen transfer as a mechanism of competition: effect of Castilleja linariaefolia pollen on seed set of Ipomopsis aggregata. Canadian Journal of Botany 78: 600–606.

[ajb21720-bib-0027] Charlebois, J. A. , and R. D. Sargent . 2017. No consistent pollinator‐mediated impacts of alien plants on natives. Ecology Letters 20: 1479–1490. 28901037 10.1111/ele.12831

[ajb21720-bib-0028] Chen, L. , B. Zhang , and Q. Li . 2017. Pollinator‐mediated selection on flowering phenology and floral display in a distylous herb Primula alpicola. Scientific Reports 7: 13157. 29030594 10.1038/s41598-017-13340-0 PMC5640686

[ajb21720-bib-0029] Conner, J. K. , and S. Rush . 1996. Effects of flower size and number on pollinator visitation to wild radish, Raphanus raphanistrum. Oecologia 105: 509–516. 28307144 10.1007/BF00330014

[ajb21720-bib-0030] Eckhart, V. M. , N. S. Rushing , G. M. Hart , and J. D. Hansen . 2006. Frequency‐dependent pollinator foraging in polymorphic Clarkia xantiana ssp. xantiana populations: implications for flower colour evolution and pollinator interactions. Oikos 112: 412–421.

[ajb21720-bib-0031] Elzinga, J. A. , A. Atlan , A. Biere , L. Gigord , A. E. Weis , and G. Bernasconi . 2007. Time after time: flowering phenology and biotic interactions. Trends in Ecology & Evolution 22: 432–439. 17573151 10.1016/j.tree.2007.05.006

[ajb21720-bib-0032] Fang, Q. , J. Gao , W. S. Armbruster , and S. Huang . 2019. Multi‐year stigmatic pollen‐load sampling reveals temporal stability in interspecific pollination of flowers in a subalpine meadow. Oikos 128: 1739–1747.

[ajb21720-bib-0033] Fang, Q. , and S.‐Q. Huang . 2013. A directed network analysis of heterospecific pollen transfer in a biodiverse community. Ecology 94: 1176–1185. 23858657 10.1890/12-1634.1

[ajb21720-bib-0034] Feinsinger, P. , H. M. Tiebout , and B. E. Young . 1991. Do tropical bird‐pollinated plants exhibit density‐dependent interactions? Field experiments. Ecology 72: 1953–1963.

[ajb21720-bib-0035] Flanagan, R. J. , R. J. Mitchell , D. Knutowski , and J. D. Karron . 2009. Interspecific pollinator movements reduce pollen deposition and seed production in Mimulus ringens (Phrymaceae). American Journal of Botany 96: 809–815. 21628236 10.3732/ajb.0800317

[ajb21720-bib-0036] Galen, C. , and T. Gregory . 1989. Interspecific pollen transfer as a mechanism of competition: consequences of foreign pollen contamination for seed set in the alpine wildflower, Polemonium viscosum. Oecologia 81: 120–123. 28312167 10.1007/BF00377020

[ajb21720-bib-0083] Gerard X. Smith Jr. Mark T. Swartz , and Rachel B. Spigler . 2021. Data from: Causes and consequences of variation in heterospecific pollen receipt in Oenothera fruticose. Dryad Digital Repository 10.5061/dryad.hqbzkh1g9 PMC9291898 34460097

[ajb21720-bib-0037] Ghazoul, J. 2005. Pollen and seed dispersal among dispersed plants. Biological Reviews 80: 413–443. 16094807 10.1017/s1464793105006731

[ajb21720-bib-0038] Gorenflo, A. , M. Van Kleunen , V. Wolters , and F. Jauker . 2017. Contrasting pollination efficiency and effectiveness among flower visitors of Malva sylvestris, Borago officianalis and Onobrychis viciifolia. Journal of Pollination Ecology 21: 62–70.

[ajb21720-bib-0039] Goulson, D. 1994. A model to predict the influence of insect flower constancy on interspecific competition between insect pollinated plants. Journal of Theoretical Biology 168: 309–314.

[ajb21720-bib-0040] Goulson, D. 2003. Bumblebees: their behaviour and ecology. Oxford University Press, Oxford, UK.

[ajb21720-bib-0041] Gross, C. L. 1992. Floral traits and pollinator constancy: foraging by native bees among three sympatric legumes. Australian Journal of Ecology 17: 67–74.

[ajb21720-bib-0042] Ha, M. K. , and C. T. Ivey . 2017. Pollinator‐mediated interactions in experimental arrays vary with neighbor identity. American Journal of Botany 104: 252–260. 28202454 10.3732/ajb.1600293

[ajb21720-bib-0043] Herrera, C. M. 1987. Components of pollinator “quality”: comparative analysis of a diverse insect assemblage. Oikos 50: 79–90.

[ajb21720-bib-0044] Herrera, C. M. 1989. Pollinator abundance, morphology, and flower visitation rate: analysis of the “quantity” component in a plant–pollinator system. Oecologia 80: 241–248. 28313114 10.1007/BF00380158

[ajb21720-bib-0045] Jordano, P. 1987. Patterns of mutualistic interactions in pollination and seed dispersal: connectance, dependence asymmetries, and coevolution. American Naturalist 129: 657–677.

[ajb21720-bib-0046] Kantsa, A. , R. A. Raguso , A. G. Dyer , J. M. Olesen , T. Tscheulin , and T. Petanidou . 2018. Disentangling the role of floral sensory stimuli in pollination networks. Nature Communications 9: 1041. 10.1038/s41467-018-03448-w PMC5847531 29531220

[ajb21720-bib-0047] Kearns, C. A. , and D. W. Inouye . 1993. Techniques for pollination biologists. University Press of Colorado, Niwot, CO, USA.

[ajb21720-bib-0048] King, C. , G. Ballantyne , and P. G. Willmer . 2013. Why flower visitation is a poor proxy for pollination: measuring single‐visit pollen deposition, with implications for pollination networks and conservation. Methods in Ecology and Evolution 4: 811–818.

[ajb21720-bib-0049] Kitamoto, N. , S. Ueno , A. Takenaka , Y. Tsumura , I. Washitani , and R. Ohsawa . 2006. Effect of flowering phenology on pollen flow distance and the consequences for spatial genetic structure within a population of Primula sieboldii (Primulaceae). American Journal of Botany 93: 226–233. 21646183 10.3732/ajb.93.2.226

[ajb21720-bib-0050] Konzmann, S. , S. Koethe , and K. Lunau . 2019. Pollen grain morphology is not exclusively responsible for pollen collectability in bumble bees. Scientific Reports 9: 4705. 30886330 10.1038/s41598-019-41262-6 PMC6423004

[ajb21720-bib-0051] Koski, M. H. , J. L. Ison , A. Padilla , A. Q. Pham , and L. F. Galloway . 2018. Linking pollinator efficiency to patterns of pollen limitation: small bees exploit the plant–pollinator mutualism. Proceedings of the Royal Society, B, Biological Sciences 285: 20180635. 10.1098/rspb.2018.0635 PMC6015843 29875304

[ajb21720-bib-0052] Koski, M. H. , G. A. Meindl , G. Arceo‐Gómez , M. Wolowski , K. A. LeCroy , and T.‐L. Ashman . 2015. Plant–flower visitor networks in a serpentine metacommunity: assessing traits associated with keystone plant species. Arthropod–Plant Interactions 9: 9–21.

[ajb21720-bib-0053] Krakos, K. N. , and M. W. Austin . 2021. Testing pollination syndromes in Oenothera (Onagraceae). Journal of Pollination Ecology 26: 52–66.

[ajb21720-bib-0054] Kunin, W. , and Y. Iwasa . 1996. Pollinator foraging strategies in mixed floral arrays: density effects and floral constancy. Theoretical Population Biology 49: 232–263. 8813024 10.1006/tpbi.1996.0013

[ajb21720-bib-0055] Kuppler, J. , M. K. Höfers , L. Wiesmann , and R. R. Junker . 2016. Time‐invariant differences between plant individuals in interactions with arthropods correlate with intraspecific variation in plant phenology, morphology and floral scent. New Phytologist 210: 1357–1368. 26840542 10.1111/nph.13858

[ajb21720-bib-0056] Liao, K. , R. W. Gituru , Y. H. Guo , and Q. F. Wang . 2011. The presence of co‐flowering species facilitates reproductive success of Pedicularis monbeigiana (Orobanchaceae) through variation in bumble‐bee foraging behaviour. Annals of Botany 108: 877–884. 21831855 10.1093/aob/mcr216 PMC3177687

[ajb21720-bib-0057] Lopezaraiza‐Mikel, M. E. , R. B. Hayes , M. R. Whalley , and J. Memmott . 2007. The impact of an alien plant on a native plant–pollinator network: an experimental approach. Ecology Letters 10: 539–550. 17542933 10.1111/j.1461-0248.2007.01055.x

[ajb21720-bib-0058] McLernon, S. M. , S. D. Murphy , and L. W. Aarssen . 1996. Heterospecific pollen transfer between sympatric species in a midsuccessional old‐field community. American Journal of Botany 83: 1168–1174.

[ajb21720-bib-0059] Méndez, M. , and A. Díaz . 2001. Flowering dynamics in Arum italicum (Araceae): relative role of inflorescence traits, flowering synchrony, and pollination context on fruit initiation. American Journal of Botany 88: 1774–1780. 21669609

[ajb21720-bib-0060] Mitchell, R. J. , R. J. Flanagan , B. J. Brown , N. M. Waser , and J. D. Karron . 2009. New frontiers in competition for pollination. Annals of Botany 103: 1403–1413. 19304814 10.1093/aob/mcp062 PMC2701753

[ajb21720-bib-0061] Montgomery, B. R. , and B. J. Rathcke . 2012. Effects of floral restrictiveness and stigma size on heterospecific pollen receipt in a prairie community. Oecologia 168: 449–458. 21833640 10.1007/s00442-011-2094-x

[ajb21720-bib-0062] Morales, C. L. , and A. Traveset . 2008. Interspecific pollen transfer: magnitude, prevalence and consequences for plant fitness. Critical Reviews in Plant Sciences 27: 221–238.

[ajb21720-bib-0063] Morales, C. L. , and A. Traveset . 2009. A meta‐analysis of impacts of alien vs. native plants on pollinator visitation and reproductive success of co‐flowering native plants. Ecology Letters 12: 716–728. 19453616 10.1111/j.1461-0248.2009.01319.x

[ajb21720-bib-0064] Moreira‐Hernández, J. I. , and N. Muchhala . 2019. Importance of pollinator‐mediated interspecific pollen transfer for angiosperm evolution. Annual Review of Ecology, Evolution, and Systematics 50: 191–217.

[ajb21720-bib-0065] Mosquin, T. 1971. Competition for pollinators as a stimulus for the evolution of flowering time as a stimulus for pollinators competition of flowering time the evolution. Oikos 22: 398–402.

[ajb21720-bib-0066] Mothershead, K. , and R. J. Marquis . 2000. Fitness impacts of herbivory through indirect effects on plant–pollinator interactions in Oenothera macrocarpa. Ecology 81: 30–40.

[ajb21720-bib-0067] Muchhala, N. , and J. D. Thomson . 2012. Interspecific competition in pollination systems: costs to male fitness via pollen misplacement. Functional Ecology 26: 476–482.

[ajb21720-bib-0068] Murphy, S. D. , and L. W. Aarssen . 1995. In vitro allelopathic effects of pollen from three Hieracium species (Asteraceae) and pollen transfer to sympatric Fabaceae. American Journal of Botany 82: 37–45.

[ajb21720-bib-0069] Ollerton, J. , R. Winfree , and S. Tarrant . 2011. How many flowering plants are pollinated by animals? Oikos 120: 321–326.

[ajb21720-bib-0070] Page, M. L. , J. L. Ison , A. L. Bewley , K. M. Holsinger , A. D. Kaul , K. E. Koch , K. M. Kolis , and S. Wagenius . 2019. Pollinator effectiveness in a composite: a specialist bee pollinates more florets but does not move pollen farther than other visitors. American Journal of Botany 106: 1487–1498. 31713237 10.1002/ajb2.1383

[ajb21720-bib-0071] Parachnowitsch, A. L. , R. A. Raguso , and A. Kessler . 2012. Phenotypic selection to increase floral scent emission, but not flower size or colour in bee‐pollinated Penstemon digitalis. New Phytologist 195: 667–675. 22646058 10.1111/j.1469-8137.2012.04188.x

[ajb21720-bib-0072] Parra‐Tabla, V. , C. Alonso , T.‐L. Ashman , R. A. Raguso , C. Albor , P. Sosenski , D. Carmona , and G. Arceo‐Gómez . 2020. Pollen transfer networks reveal alien species as main heterospecific pollen donors with fitness consequences for natives. Journal of Ecology 108: 1–13.

[ajb21720-bib-0073] Pleasants, J. M. 1980. Competition for bumblebee pollinators in Rocky Mountain plant communities. Ecology 61: 1446–1459.

[ajb21720-bib-0074] Primack, R. B. , and J. A. Silander . 1975. Measuring the relative importance of different pollinators to plants. Nature 255: 143–144.

[ajb21720-bib-0075] R Core Team . 2020. R: A language and environment for statistical computing. R Foundation for Statistical Computing, Vienna, Austria. Website: https://www.R-project.org

[ajb21720-bib-0076] Randall, J. L. , and K. W. Hilu . 1990. Interference through improper pollen transfer in mixed stands of Impatiens capensis and I. pallida (Balsaminaceae). American Journal of Botany 77: 939–944.

[ajb21720-bib-0077] Randle, A. M. , R. B. Spigler , and S. Kalisz . 2018. Shifts to earlier selfing in sympatry may reduce costs of pollinator sharing. Evolution 72: 1587–1599. 10.1111/evo.13522 29917223

[ajb21720-bib-0078] Rathcke, B. J. 1983. Competition and facilitation among plants for pollination. Pollination Biology, 205–329. Academic Press, NY, NY, USA.

[ajb21720-bib-0079] Rhodes, M. K. , J. B. Fant , and K. A. Skogen . 2017. Pollinator identity and spatial isolation influence multiple paternity in an annual plant. Molecular Ecology 26: 4296–4308. 28334485 10.1111/mec.14115

[ajb21720-bib-0080] Roll, J. , R. J. Mitchell , R. J. Cabin , and D. L. Marshall . 1997. Reproductive success increases with local density of conspecifics in a desert mustard. Conservation Biology 11: 738–746.

[ajb21720-bib-0081] Sahli, H. F. , and J. K. Conner . 2007. Visitation, effectiveness, and efficiency of 15 genera of visitors to wild radish, Raphanus raphanistrum (Brassicaceae). American Journal of Botany 94: 203–209. 21642222 10.3732/ajb.94.2.203

[ajb21720-bib-0082] Silander, J. , and R. Primack . 1978. Pollination intensity and seed set in the evening primrose (Oenothera fruticosa). American Midland Naturalist 100: 213–216.

[ajb21720-bib-0084] Smithson, A. 2001. Pollinator preference, frequency dependence, and floral evolution. In L. Chittka and J. D. Thomson [eds.], Cognitive ecology of pollination: animal behavior and floral evolution, 237–258. Cambridge University Press, Cambridge, UK.

[ajb21720-bib-0085] Spigler, R. B. , and S. M. Chang . 2008. Effects of plant abundance on reproductive success in the biennial Sabatia angularis (Gentianaceae): spatial scale matters. Journal of Ecology 96: 323–333.

[ajb21720-bib-0086] Stone, G. N. , P. Willmer , and J. Alexandra Rowe . 1998. Partitioning of pollinators during flowering in an African Acacia community. Ecology 79: 2808–2827.

[ajb21720-bib-0087] Stout, J. C. , J. A. Allen , and D. Goulson . 1998. The influence of relative plant density and floral morphological complexity on the behaviour of bumblebees. Oecologia 117: 543–550. 28307680 10.1007/s004420050691

[ajb21720-bib-0088] Suárez‐Mariño, A. , G. Arceo‐Gómez , P. Sosenski , and V. Parra‐Tabla . 2019. Patterns and effects of heterospecific pollen transfer between an invasive and two native plant species: the importance of pollen arrival time to the stigma. American Journal of Botany 106: 1308–1315. 31553505 10.1002/ajb2.1361

[ajb21720-bib-0089] Thomson, J. D. , B. J. Andrews , R. C. Plowright . 1981. The effect of a foreign pollen on ovule fertilization in Diervilla lonicera (Caprifoliaceae). New Phytologist 90: 777–783.

[ajb21720-bib-0090] Thomson, J. D. , H. F. Fung , and J. E. Ogilvie . 2019. Effects of spatial patterning of co‐flowering plant species on pollination quantity and purity. Annals of Botany 123: 303–310. 29947735 10.1093/aob/mcy120 PMC6344345

[ajb21720-bib-0091] Tur, C. , A. Sáez , A. Traveset , and M. A. Aizen . 2016. Evaluating the effects of pollinator‐mediated interactions using pollen transfer networks: evidence of widespread facilitation in south Andean plant communities. Ecology Letters 19: 576–586. 26991916 10.1111/ele.12594

[ajb21720-bib-0092] Tur, C. , B. Vigalondo , K. Trøjelsgaard , J. M. Olesen , and A. Traveset . 2014. Downscaling pollen‐transport networks to the level of individuals. Journal of Animal Ecology 83: 306–317. 24107193 10.1111/1365-2656.12130

[ajb21720-bib-0093] Vázquez, D. P. , and M. A. Aizen . 2004. Asymmetric specialization: a pervasive feature of plant–pollinator interactions. Ecology 85: 1251–1257.

[ajb21720-bib-0094] Waser, N. M. 1978. Competition for hummingbird pollination and sequential flowering in two Colorado wildflowers. Ecology 59: 934–944.

[ajb21720-bib-0095] Waser, N. M. 1986. Flower constancy: definition, cause, and measurement. American Naturalist 127: 593–603.

[ajb21720-bib-0096] Waser, N. M. , L. Chittka , M. V Price , N. M. Williams , and J. Ollerton . 1996. Generalization in pollination systems, and why it matters. Ecology 77: 1043–1060.

